# Scripted spot removal in PBS proton therapy planning

**DOI:** 10.1002/acm2.13491

**Published:** 2021-12-10

**Authors:** Samantha G. Hedrick, Bryant Walker, Bart Morris, Scott Petro, Marc Blakey

**Affiliations:** ^1^ Provision CARES Proton Therapy Center Knoxville Tennessee USA

**Keywords:** outcomes, PBS, scripting

## Abstract

**Background:**

It is well known in proton therapy that the relative biological effectiveness (RBE) is not constant across the entire Bragg peak, with higher RBE at the distal end of the Bragg peak due to higher linear energy transfer (LET). Treatment planning systems are moving toward LET optimization to mitigate this potentially higher biological impact at a track end. However, using a simple script, proton users can begin to simulate this process by deleting spots from critical structures during optimization. In most cases, nominal target coverage and plan robustness remain satisfactory.

**Methods:**

In our clinic, we developed a script that allows the user to delete spots in all organs at risk (OARs) of interest for one or more treatment beams. The purpose of this script is to potentially reduce side effects by eliminating Bragg peaks within OARs. The script was first used for prostate patients where spots in the rectum and sigmoid, outside of the overlap with the target, were deleted. We then began to use the script for head and neck (H&N) and breast/chestwall patients to reduce acute side effects of the skin by removing spots in a 0.5‐cm skin rind.

**Conclusions:**

By utilizing a simple script for deleting spots in critical structures, we have seen excellent clinical results thus far. We have noted reduced skin reactions for nearly all H&N and breast patients.

## 1 INTRODUCTION

Currently, most proton therapy centers plan with a constant relative biological effectiveness (RBE) of 1.1, that is, the biological dose for proton therapy is 10% higher than the physical dose.^1^, ^2^, ^3^ However, it is well known that the RBE is not constant across the entire Bragg peak, with higher RBE at the distal end of the Bragg peak due to higher linear energy transfer (LET).^4^ RBE values could be as high as 1.4–1.7, possibly even up to 3.0, in the fall‐off of the Bragg peak.^5^, ^6^, ^7^, ^8^ Typically, most proton therapy centers will avoid ending all treatment beams on a serial organ, such as the brainstem or spinal cord, to reduce the uncertainty of a higher biological dose to these organs at risk (OAR).^9^ This was highly relevant for scattering delivery systems because the highest weighted spots were always placed at the distal end of the spread‐out Bragg peak (SOBP). In the modern era of pencil beam scanning (PBS), high weighted spots are not necessarily always at the distal end of the beam because the SOBP varies based on patient anatomy. Additionally, with the use of multi‐field optimization (MFO) treatment planning, a treatment beam does not necessarily have a uniform dose distribution. Proton spots can be placed anywhere around the target, including in OARs, and there could be a higher biological impact than what is reflected in the dose visualization when applying a constant RBE.

Treatment planning systems are moving toward LET optimization, which would optimize not only target coverage and OAR sparing, but also the biological impact of spot placement. Ideally, spot placement would be optimized such that high weighted spots are focused within the target and away from critical structures. However, this is not yet commercially and clinically available in the United States, so users can mimic this effect by manually optimizing spot placement.

**FIGURE 1:**
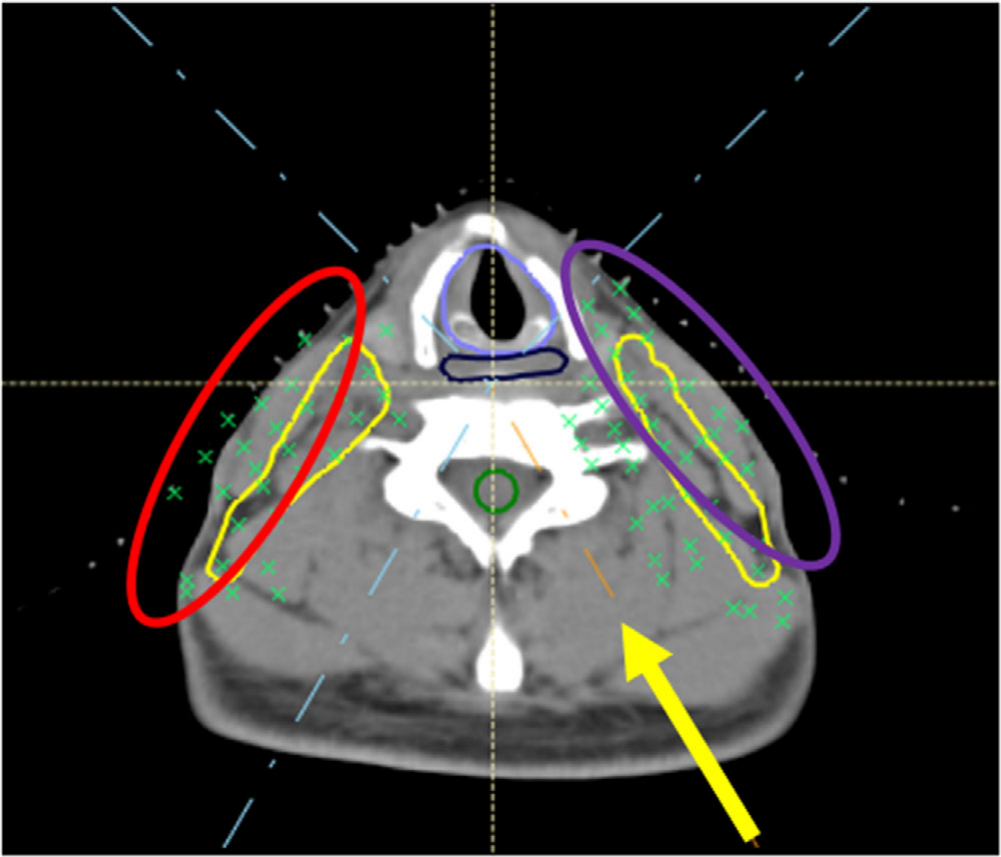
Typical head and neck (H&N) spots distribution from a left posterior oblique beam, indicated by yellow arrow. Circles identify the areas of spots in the skin due to ending anteriorly (red circle) and skimming (purple circle)

There are some features within current treatment planning systems to control spot placement. Some will allow the user to choose a minimum depth at which a spot can be placed, which can control proximal skin sparing, but cannot control spots beyond that depth. For example, if the user wants to spare a 0.5‐cm skin rind, they can choose a minimum depth of 0.5 cm to place spots. This method is ideal for skin sparing when a beam enters through the skin en face. However, when a beam enters tangentially through the skin, the 0.5‐cm minimum depth no longer correlates with a 0.5‐cm skin rind due to obliquity, as shown in Figure 1 in the purple circle. Additionally, this method cannot control spots that end on the skin, as shown in Figure 1 in the red circle. Some treatment planning systems will allow the user to choose a contour to “avoid,” where a beam cannot place spots through or within this structure, which can effectively control spot placement in an OAR. However, there are situations in which a user would want to deliver spots through the OAR, ending distal to the structure rather than in the structure, but this is not feasible with an “avoid” technique. Additionally, some treatment planning systems allow manual spot editing, in which the user can choose an individual spot to delete, but this is tedious and time consuming, and there is no commercial method for deleting spots in an entire OAR or contours.

In our clinic, we have developed a simple script within our treatment planning system that allows the user to delete spots in a contour for one or more treatment beams. The purpose of this script is to potentially reduce side effects by eliminating Bragg peaks within OARs. The script was first used for prostate patients where spots in the rectum and sigmoid, outside of the overlap with the target, were deleted. Accordingly, we then began to use the script for head and neck (H&N) and breast patients to reduce acute side effects in the skin by removing spots in a 0.5‐cm skin rind.

## 2 METHODS

A script was developed in IronPython and implemented in RayStation V8a (RaySearch Americas). The user can choose one or more contours in which to remove spots, designated as the “removal contour.” The user can also choose if they want to keep spots in the region where the target overlaps with the removal contour. The script uses each slice of the removal contour to create a 2‐D polygon. Then it cycles through each of the spot coordinates with the same z‐coordinate value within a standard deviation to ensure that all coordinates are captured, even if it falls between CT slices. A ray casting algorithm is used to determine if the spot is within the 2‐D polygon. The algorithm casts a trace from the spot coordinate through a line segment. This is repeated for all line segments that make up the polygon for that point. The algorithm checks if the y‐coordinate of the point is less than or greater than the vertices of the line segment to determine if the trace intersects the polygon. If the sum of the intersections is an even number, the spot is outside the contour and is ignored. If the sum of the intersections is an odd number, the spot is inside the contour and is deleted. This process is repeated for all points with common z‐coordinates and is then repeated for all slices.

To integrate the script into our planning process, the treatment planner need only run a single iteration to allow the optimizer to place the spots. The script is used to delete spots from the selected removal contour, and then the user can continue the optimization. The optimizer does not change the position of the remaining spots; it simply redistributes the spot weights to meet the planning objectives.

**FIGURE 2:**
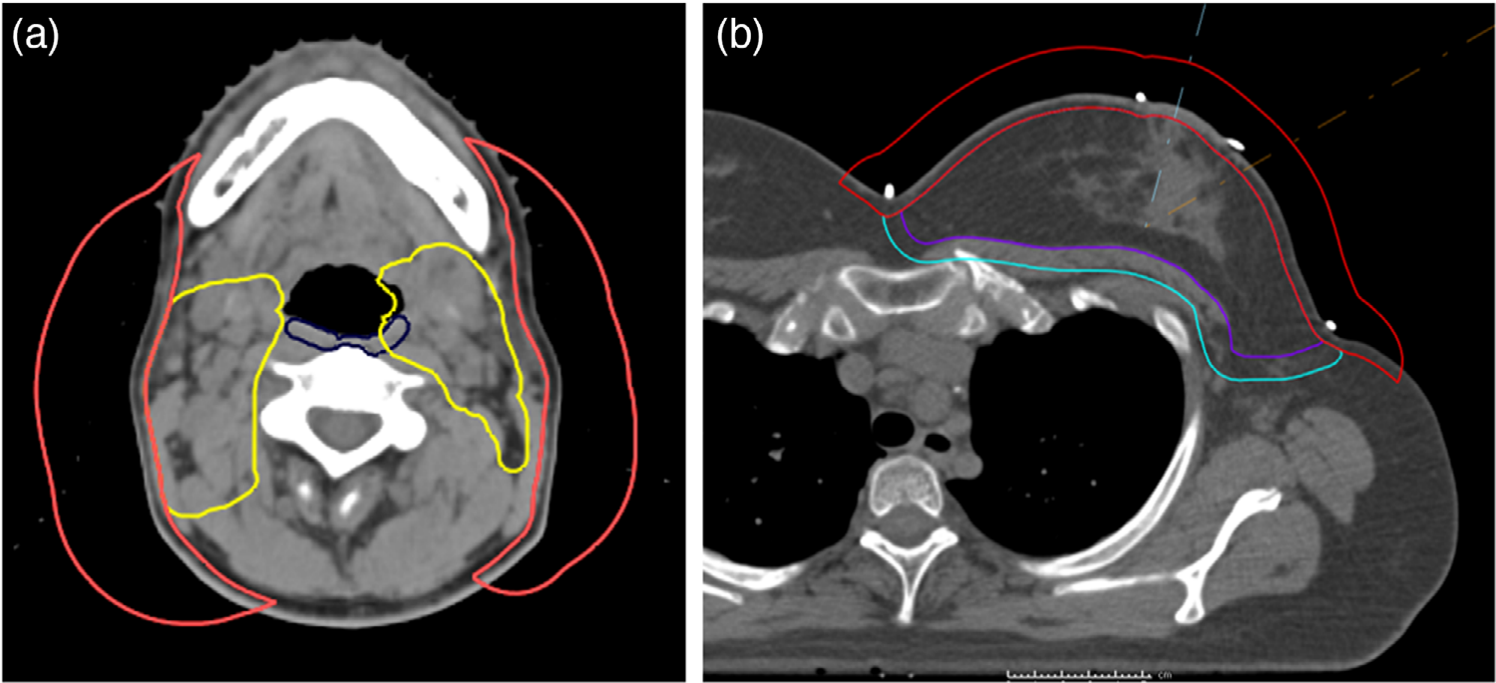
“SkinSpots” contour (red) for (a) head and neck (H&N) and (b) breast plans

For prostate patients in this study, the user chose the rectum and sigmoid as the removal contour, keeping spots in the overlap with the PTV. For H&N and breast/chestwall patients, a “SkinSpots” contour was created using the following algebra: Target+2 cm – “Skin” contracted 0.5 cm, shown in Figure 2. This created a contour that includes the 0.5‐cm skin rind plus an expansion into the air to eliminate spots in the skin and at the interface of the patient and air.

## 3 RESULTS

At the time of writing, our planning team has implemented this technique on 14 prostate patients, 18 breast and chestwall patients, and 21 H&N patients. The script requires between 30 min and 2 h to run, depending on the size of the removal contour, number of spots, and computing speed.

### 3.1 Prostate

**FIGURE 3:**
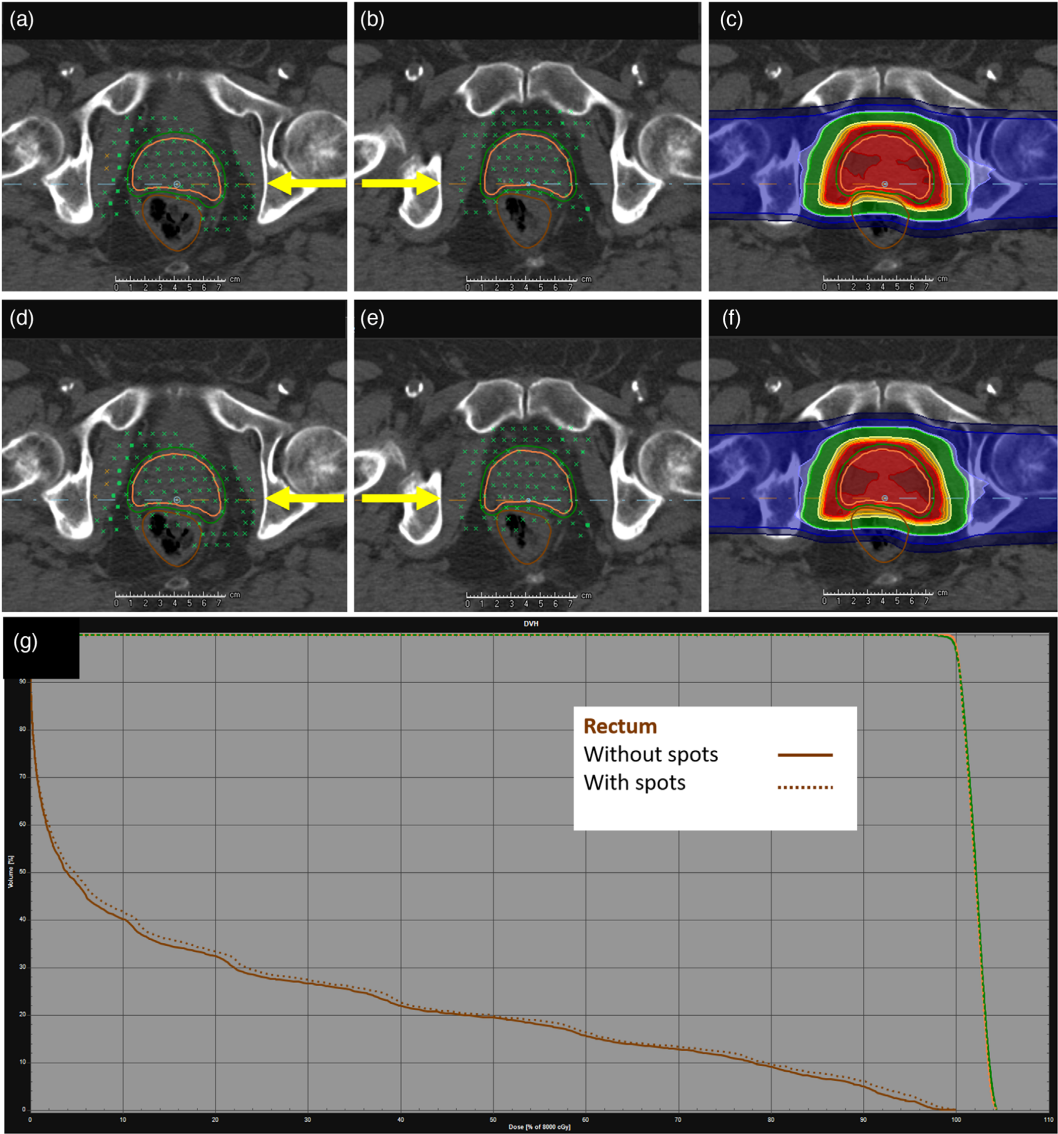
Example of spot removal script process for a two‐field prostate case. (a and b) right and left lateral beams with spots removed. (c) Isodose for plan with spots removed. (d and e) Right and left lateral beams with spots. (f) Isodose for plan with spots in rectum. Yellow arrows indicate beam direction. (g) Dose‐volume histogram (DVH) comparison. Rectum – brown, without spots – solid, with spots – dashed

For a typical intermediate risk prostate patient, removing spots out of the rectum and sigmoid minus the target resulted in the deletion of about 150 spots, which accounts for 4%–5% of the total spots. As shown in Figure 3, both beams have spots removed in the rectum (brown contour) minus overlap with the target. Overall, for a sample patient planned to 80 cobalt gray equivalent (CGE), the change in the nominal dose is minimal, as indicated in Figure 3C,F. There is typically a slight decrease in mean dose to the rectum and sigmoid, but it is not necessarily significant. In all prostate cases, there is no impact on target coverage or plan robustness compared to a plan in which spots were not edited. The robustness comparison for a sample prostate patient is compared in Table 1 for the rectum maximum dose, clinical tumor volume (CTV) maximum dose, and CTV D95%. The nominal (unperturbed) value for each plan, both with and without spots, is presented with the range of perturbed values (nominal [minimum perturbed and maximum perturbed]). The 12 perturbations included are 0.3‐cm shifts in +/− X, Y, and Z directions, +/− 3.5% range uncertainty, and +/− 3° roll and yaw. Our prostate plans are typically planned using single field optimization (SFO), so we do not require analysis of independent beam shifts. On average, across the 12 perturbations, the CTV D95% does not change by more than 0.47% (0.38 CGE) without spots and 0.44% (0.35 CGE) spots.

**TABLE 1:** Prostate robust analysis comparison for a sample patient, planned to 80 CGE for the prostate CTV. Twelve perturbations were analyzed. The nominal value (unperturbed) is presented for each metric, with the range of perturbed values (minimum, maximum)

Prostate robust analysis comparison Structure	Without spots	With spots
Rectum (Dmax)	80.00 CGE (78.97, 83.85)	80.20 CGE (79.10, 85.20)
CTV (Dmax)	83.49 CGE (83.42, 87.27)	83.50 CGE (83.38, 87.13)
CTV D95%	80.19 CGE (78.41, 80.28)	80.20 CGE (78.43, 80.30)

### 3.2 Breast

**FIGURE 4:**
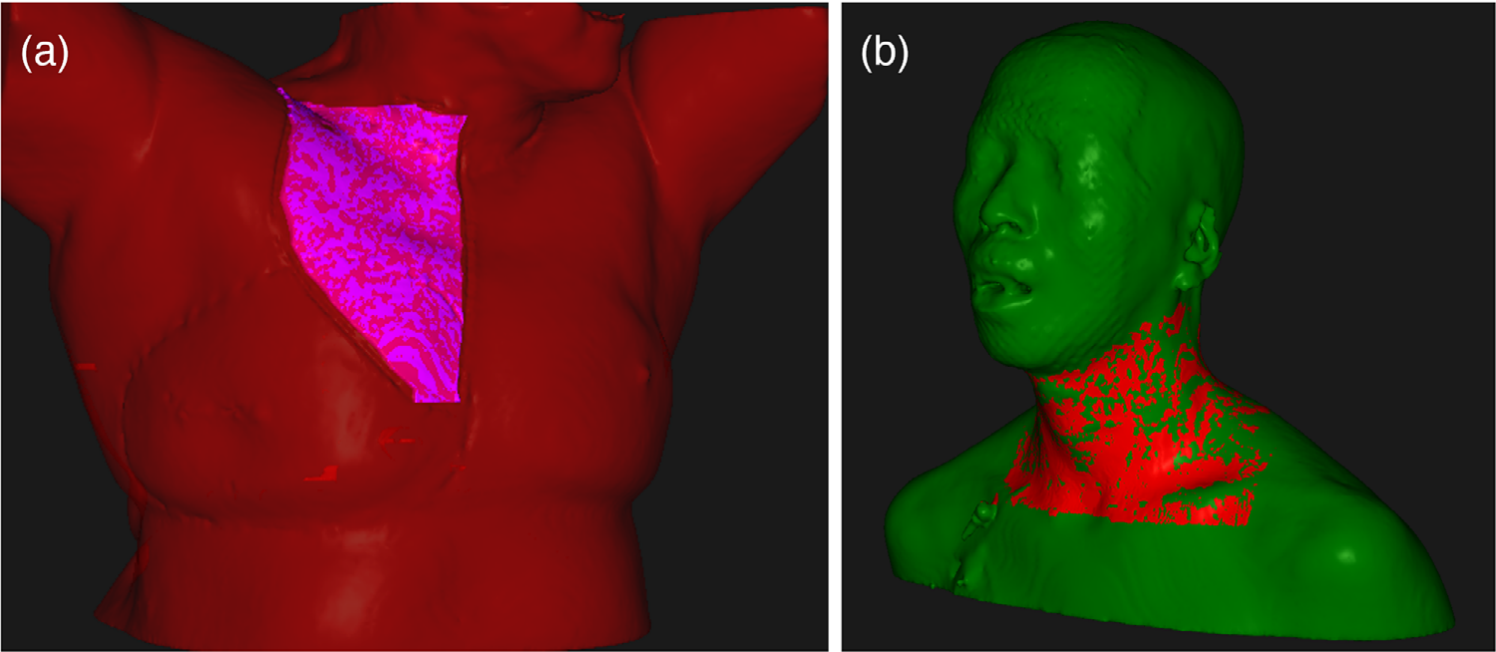
Example of typical skin reaction locations for breast (left) and H&N (right) patients before spot removal technique. For the breast patient, the area of typical skin reaction is shaded in pink. For the H&N patient, the area of typical skin reaction is shaded in red

In breast patients, the medial portion of the chest on the ipsilateral side is the area of most adverse skin reactions, as seen in Figure 4A. When the beam is en face to the skin, in the apex of the breast, the inherent minimum spot depth controls the spot placement well, so we do not typically see adverse reactions. In this medial and lateral region, however, the spot depth is not well controlled because en face beams are skimming tangentially along the medial and lateral portions of the breast. Additionally, a more lateral beam is treating through the breast and ending on the medial skin. Our script can remove these spots, regardless of beam direction.

**FIGURE 5:**
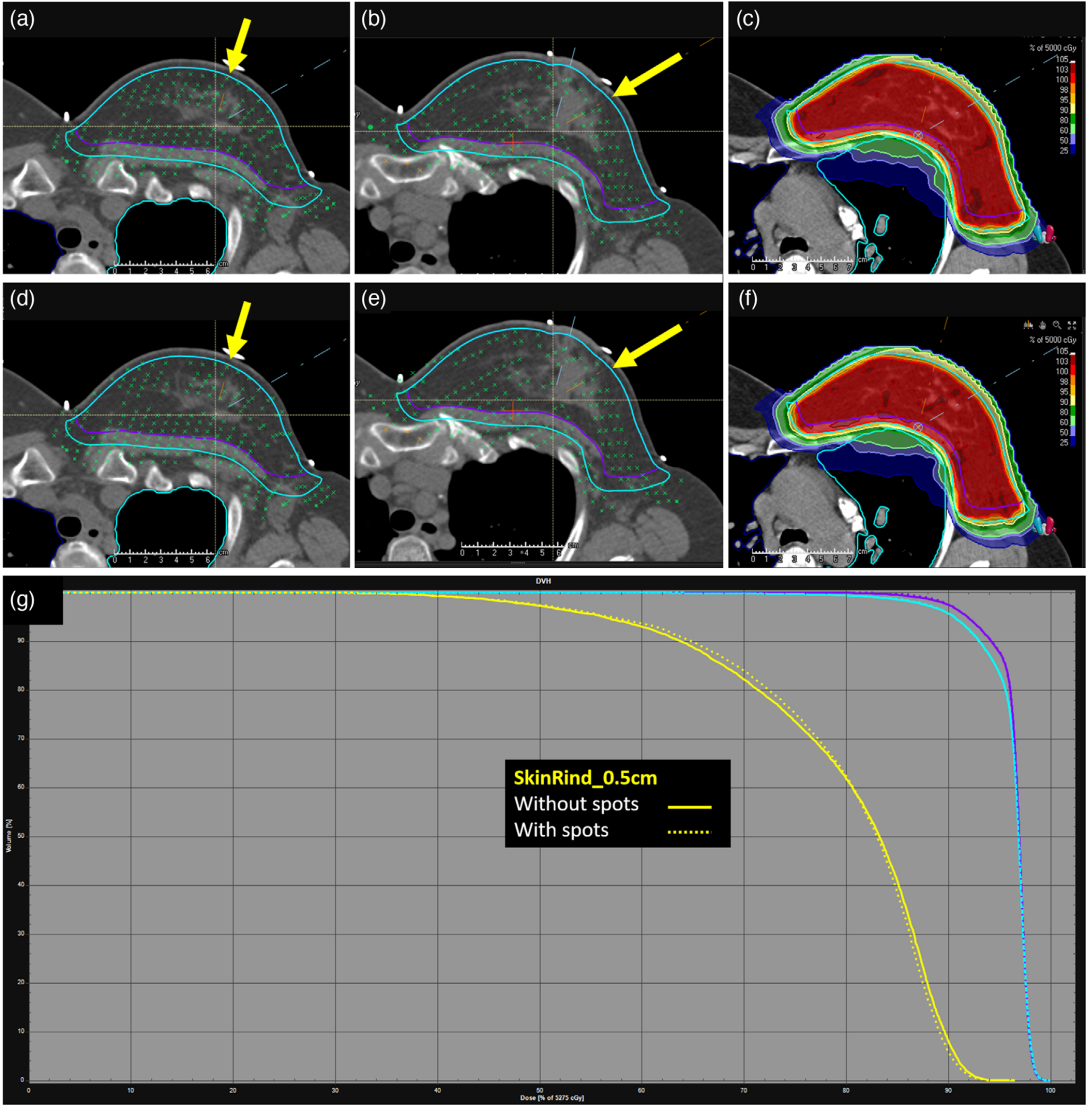
Example of spot removal script process for a two‐field breast case. (a and b) Medial and lateral beams with spots removed. (c) Isodose for plan with spots removed. (d and e) Medial and lateral beams with spots. (f) Isodose for plan with spots in skin rind. Yellow arrows indicate beam direction. (g) Dose‐volume histogram (DVH) comparison. SkinRind_0.5 cm – yellow, without spots – solid, with spots – dashed

For a typical breast patient where we remove spots from the 0.5‐cm skin rind, the script deletes about 500 spots, which accounts for 3%–5% of the total spots. Spots in the apex of the breast are mostly unchanged, while the spots from the medial and lateral aspects of the breast are primarily deleted, as seen in Figure 5A,B,D,E.

For a sample patient, planned for 50 CGE to the whole Breast_CTV, the isodoses are very similar between the two plans, as indicated in Figure 5C,F. The 0.5‐cm skin rind within the Breast_PTV was contoured and is shown in Figure 5G, in yellow. There is very little impact on the dose‐volume histogram (DVH) for the skin rind when deleting spots. For all breast cases, there is no impact on target coverage or plan robustness compared to a plan in which spots were not edited. The robustness comparison for a sample breast patient is compared in Table 2 for the heart maximum dose, Breast_CTV maximum dose, and Breast_CTV D95%. The nominal (unperturbed) value for each plan, both with and without spots, is presented with the range of perturbed values (nominal [minimum perturbed and maximum perturbed]). The 12 perturbations included 0.3‐cm shifts in +/− X, Y, and Z directions, +/− 3.5% range uncertainty, and +/− 3° roll and yaw. Our breast plans are typically SFO, so we do not require analysis of independent beam shifts. On average, across the 12 perturbations, the Breast_CTV D95% does not change by more than 1.03% (0.50 CGE) without spots and 0.87% (0.42 CGE) with spots.

**TABLE 2:** Breast robust analysis comparison for a sample patient, planned to 50 CGE for the whole breast. Twelve perturbations were analyzed. The nominal value (unperturbed) is presented for each metric, with the range of perturbed values (minimum, maximum)

Breast robust analysis comparison Structure	Without spots	With spots
Heart (Dmax)	37.03 CGE (28.04, 43.02)	35.50 CGE (27.40, 42.86)
Breast_CTV (Dmax)	52.73 CGE (52.56, 53.31)	52.75 CGE (52.52, 53.16)
Breast_CTV D95%	48.46 CGE (46.96, 48.95)	48.44 CGE (47.30, 48.86)

### 3.3 H&N

In the case of an H&N patient, in which we are using four beams to treat in a modified X arrangement, there is skimming of all four beams on the lateral edge of the neck. The sides and anterior portion of the neck are where we are seeing the most adverse skin reactions, as seen in Figure 4B. Additionally, there are often cases in which spots are ending distally on the skin, and this cannot be controlled during optimization. In the case of H&N, posterior beams treat through the neck and end on anterior skin while anterior beams end on the lateral portions of the neck. In these cases, the script can remove all skin rind spots, potentially reducing side effects.

**FIGURE 6:**
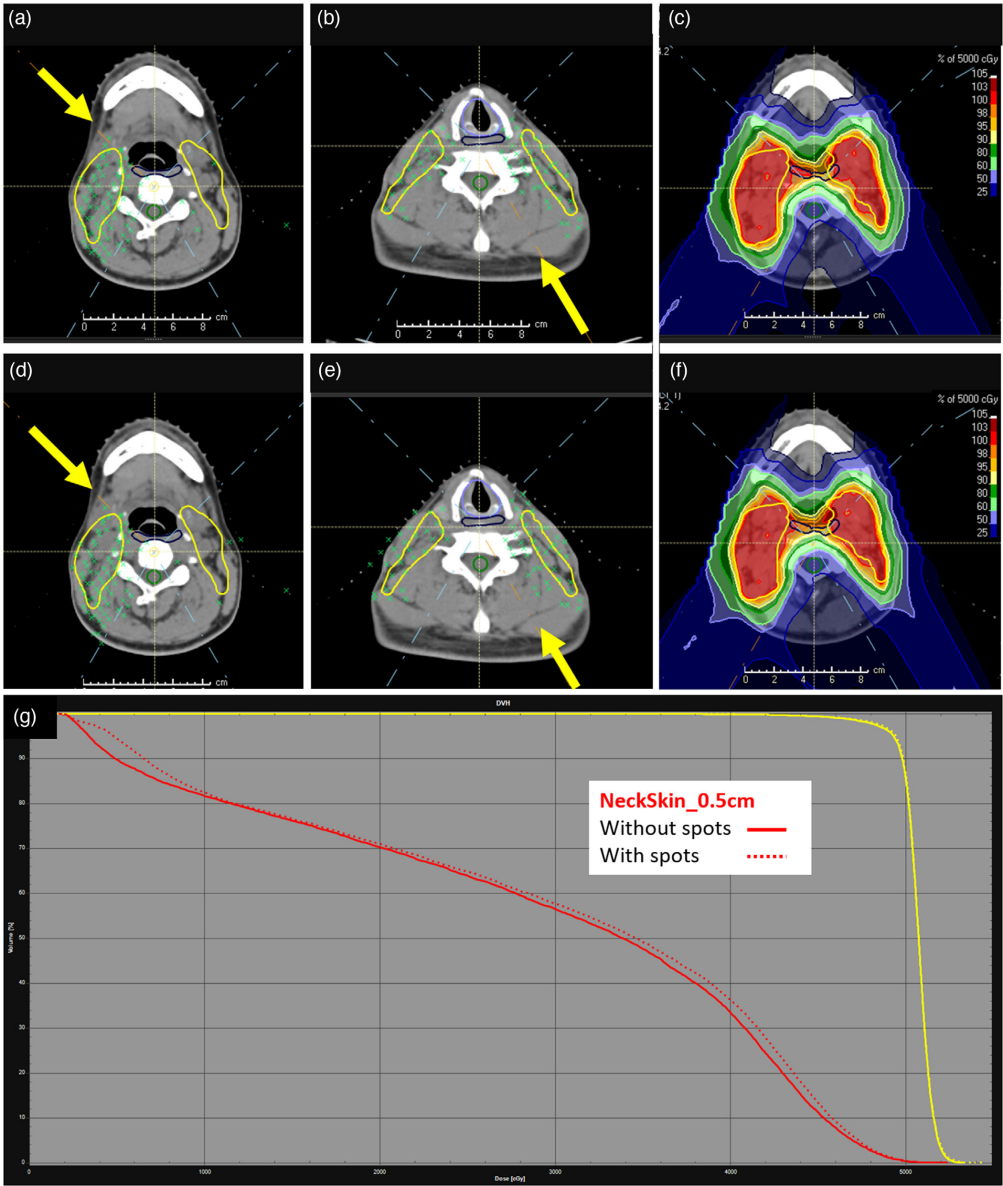
Example of spot removal script process for a four‐field head and neck (H&N) case. (a and b) right anterior oblique (RAO) and left posterior oblique (LPO) beams with spots removed. (c) Isodose for plan with spots removed. (d and e) RAO and LPO with spots. (f) Isodose for plan with spots in skin rind. Yellow arrows indicate beam direction. (g) Dose‐volume histogram (DVH) comparison. NeckSkin_0.5 cm – red, without spots – solid, with spots – dashed

For a typical H&N patient, deleting spots from a 0.5‐cm skin rind removes about 1000 spots, which accounts for 9%–10% of the total spots. As seen in Figure 6A,B,D,E, spots are deleted both in the proximal and distal skin rind for each of the four treatment beams. For a sample H&N patient, planned to 50 CGE for the initial phase of a sequential 70 CGE plan, there is little impact on the isodoses, as shown in Figure 6C,F. However, there is a small improvement to the DVH for a 0.5‐cm skin rind, contoured only in the neck and supraclavicular region. For all H&N cases, there is no impact on target coverage or plan robustness compared to a plan in which spots were not edited. The robustness comparison for a sample H&N patient is compared in Table 3 for the BrainStem maximum dose, SpinalCord maximum dose, CTV50 maximum dose, and CTV50 D95%. The nominal (unperturbed) value for each plan, both with and without spots, is presented with the range of perturbed values (nominal [minimum perturbed and maximum perturbed]). The 18 perturbations included 0.3‐cm shifts in +/− X, Y, and Z directions, +/− 3.5% range uncertainty, +/− 3° roll and yaw, and 0.3‐cm shifts of a single beam in +/− X, Y, and Z directions. Because our H&N plans are MFO, we analyze independent beam shifts to evaluate gradients. On average, across the 18 perturbations, the CTV50 D95% does not change by more than 1.57% (0.77 CGE) without spots and 1.54% (0.76 CGE) spots.

**TABLE 3:** Head and neck (H&N) robust analysis comparison for a sample patient, planned to 50 CGE for the CTV, as the first phase of a sequential H&N plan. Eighteen perturbations were analyzed. The nominal value (unperturbed) is presented for each metric, with the range of perturbed values (minimum, maximum)

H&N robust analysis comparison		
Structure	Without spots	With spots
BrainStem (Dmax)	35.44 CGE (30.95, 40.08)	35.32 CGE (30.69, 40.22)
SpinalCord (Dmax)	29.15 CGE (26.50, 32.57)	25.45 CGE (23.21, 29.64)
CTV50 (Dmax)	53.28 CGE (53.28, 57.55)	54.46 CGE (53.82, 59.86)
CTV50 D95%	49.30 CGE (47.79, 49.30)	49.44 CGE (47.73, 49.44)

### 3.4 Clinical results

Currently, we are in the process of collecting objective outcomes data for these patients. Since the implementation of this technique, physicians and therapists have reported improved skin reactions for H&N and breast patients, with less erythema and less dry and moist desquamation. We have not yet seen an acute impact on prostate patients.

## 4 DISCUSSIONS

The goal of this work is to improve outcomes for patients by optimizing the biological impact of proton therapy with the removal of spots from critical structures. The theory that higher biological impact occurs at the point at which a spot is delivered is the motivation to remove spots from OARs. The impact of LET is not modeled in our treatment planning system, so we are awaiting further clinical outcomes to truly evaluate the impact of this work.

We define robustness as the plan's ability to maintain coverage and OAR sparing not only on robust analysis performed before the patient starts treatment, but also on quality assurance CT analysis that is performed periodically while the patient is under beam. When we started this process, we anticipated that plans would be less robust after deleting spots. However, for prostate cases, we have seen no impact on plan robustness. In the cases of breast and H&N, we saw slightly increased hotspots near the skin, likely due to increased gradients without spots in the skin, but these hotspots were well within tolerance and do not seem to have negatively impacted skin reactions. Additionally, we anticipated seeing a distribution of higher weighted spots around the structures in which spots were deleted to make up for the potential loss of coverage due to spot deletion. However, after continuing the optimization after deletion, the optimizer was able to regain lost coverage, with improved OAR sparing, without needing to heavily weight the remaining spots.

In the cases of H&N and breast/chestwall, in which we are deleting spots out of the 0.5‐cm skin rind, we are already seeing reduced skin reactions that will be quantified in future work. For most of these patients, there is no overlap with the target and the 0.5‐cm skin rind, so there is no reason to place a spot in the skin. Our treatment planning system allows the user to choose a minimum depth at which a spot will be placed. However, this is only relevant for the en face portion of the patient; therefore spots can still be delivered in the skin rind at the tangential portions of the beam entrance.

In the case of prostate treatment, an avoidance structure could be placed on the rectum and sigmoid, but this prevents a beam from placing spots in, or distal to, the avoidance structure. Utilizing the script allows the beam to treat with spots distal to the rectum and sigmoid, if needed for target coverage, while removing spots within the OARs.

Overall, we have seen excellent clinical results thus far, with reduced skin reactions for nearly all H&N and breast patients using this spot removal method. We are collecting the clinical outcomes data for these patients and will publish the results in follow‐up work. We are currently evaluating the ideal skin contour thickness in which to delete spots, determining if a larger skin rind is feasible, without sacrificing coverage or robustness. We are also expanding the use of this work in H&N patients to evaluate the impact of spot removal in other OARs, such as oral cavity, esophagus, and pharyngeal constrictors, to reduce mucositis and swallowing dysfunction.

We are also working on enhancements to the script itself. Our spot size is fairly large, at 1.6‐cm FWHM, and the script only deletes a spot if the centroid of the spot is within the spot‐removal contour. We are evaluating if the expansion of the desired contour should be larger to account for this, or if the script can expand the coordinates of each spot. The run time, while not hugely impactful to our workflow, is still too long. We are evaluating the impact of our hardware and server environment to determine if the script can be faster if run locally.

## 5 CONCLUSIONS

Through a simple scripting technique, proton therapy users can potentially reduce patient side effects by minimizing or eliminating proton spots in critical structures, such as the skin, without sacrificing target coverage or robustness.

## CONFLICT OF INTEREST

The authors declare that there is no conflict of interest that could be perceived as prejudicing the impartiality of the research reported.

## AUTHOR CONTRIBUTIONS

Samantha G. Hedrick and Bryant Walker wrote the script. Samantha G. Hedrick, Bart Morris, Scott Petro, and Marc Blakey developed the technique. Bart Morris collected outcome data.

## Supporting information

Supporting Information

## Data Availability

The data that support the findings of this study are available from the corresponding author upon reasonable request.
