# Supplementary material for: Scripted spot removal in PBS proton therapy planning
Source: J Appl Clin Med Phys. 2021 Dec 10;23(2):e13491. doi: 10.1002/acm2.13491 (PMC8833280; doi:10.1002/acm2.13491)
Supplement: Supplementary file 1 — Supporting Information [file ACM2-23-e13491-s001.docx]

Scripted spot removal in PBS proton therapy planning

Samantha G Hedrick

Bryant Walker

Bart Morris

Scott Petro

Marc Blakey

Samantha G Hedrick, PhD
Provision CARES Proton Therapy Center

6450 Provision Cares Way

Knoxville, TN 37909

[samantha.hedrick@provisionproton.com](mailto:samantha.hedrick@provisionproton.com)

Bryant Walker, PhD
Provision CARES Proton Therapy Center

6450 Provision Cares Way

Knoxville, TN 37909

[bwalke18@gmail.com](mailto:bwalke18@gmail.com)

Bart Morris, MS
Provision CARES Proton Therapy Center

6450 Provision Cares Way

Knoxville, TN 37909

[bart.morris@provisionproton.com](mailto:bart.morris@provisionproton.com)

Scott Petro, MS
Provision CARES Proton Therapy Center

6450 Provision Cares Way

Knoxville, TN 37909

[scott.petro@provisionproton.com](mailto:scott.petro@provisionproton.com)

Marc Blakey, MS
Provision CARES Proton Therapy Center

6450 Provision Cares Way

Knoxville, TN 37909

[marc.blakey@provisionproton.com](mailto:marc.blakey@provisionproton.com)

**Running title:** Scripted spot removal in PBS planning

**Author contribution statement:**

Samantha G Hedrick and Bryant Walker wrote the script. Samantha G Hedrick, Bart Morris, Scott Petro, and Marc Blakey developed the technique. Bart Morris collected outcome data.
